# A qualitative interview study exploring the lived experiences of adults, adolescents, and children with chronic inducible cold urticaria

**DOI:** 10.1186/s41687-025-00970-6

**Published:** 2025-12-06

**Authors:** Ashna Alladin, Isabelle Guillemin, Chien-Chia Chuang, Jingdong Chao, Marieke Krol, Efstathios Zikos, Renata Martincova, Ella Brookes

**Affiliations:** 1 IQVIA Patient Centered Solutions, Munich, Germany; 2 IQVIA Patient Centered Solutions, Lyon, France; 3 Sanofi Health Economics and Value Assessment, Cambridge, MA USA; 4 Regeneron Pharmaceuticals Inc., Sleepy Hollow, NY USA; 5 IQVIA Patient Centered Solutions, Amsterdam, Netherlands; 6 Sanofi, Gentilly, France; 7 Sanofi, Prague, Czech Republic; 8 Patient Informed Development & Health Value Translation, Sanofi, 410 Thames Valley Park Drive, Reading, RG6 1PT UK

**Keywords:** Cold urticaria, ColdU, Chronic inducible cold urticaria, Disease conceptual model, Qualitative patient interviews, Concept elicitation, Adults, Adolescents and children, HRQoL

## Abstract

**Background:**

Chronic inducible cold urticaria (ColdU) is characterized by itchy wheals (hives) with or without angioedema induced by cold exposure. It severely impacts patients’ health-related quality of life (HRQoL). Qualitative research describing the burden of ColdU from direct patients’ voice remains sparse. The study aims to explore the patients’ ColdU experience by identifying signs, symptoms, and associated impacts, and to develop the first conceptual model for ColdU from the patients’ perspective.

**Methodology:**

One-to-one telephone concept elicitation (CE) interviews were conducted with patients with ColdU between 2022 and 2023. Participants were asked open-ended questions about their experience of the signs, symptoms and impacts and the most and least bothersome aspects of their disease.

**Results:**

Eight adults and 5 adolescent participants interviewed reported 22 ColdU-related symptoms/signs and 32 impacts. Six children/caregiver dyads and 1 caregiver of a 2-year-old child with ColdU reported 17 ColdU-related symptoms/signs and 19 impacts. Patient experience of symptoms and impacts was similar across all age groups, with hives and itch reported most frequently as the most bothersome symptoms. Similarly, for impacts, all participants reported impairment in activities of daily living (e.g., not taking part in hobbies, wearing warm clothes). Two conceptual models of patients’ experience of ColdU (one for adult/adolescent patients and one for pediatric patients) were developed.

**Conclusions:**

This study is the first to report the ColdU experiences directly from patients. It identifies hives and itch as being the core symptoms and describes the debilitating nature of ColdU and its substantial impact on the HRQoL of all age ranges.

## Background

Chronic inducible cold urticaria, commonly known as cold urticaria (ColdU), represents a distinctive subset within the spectrum of chronic inducible urticaria by manifesting uniquely in individuals exposed to cold stimuli. The stimuli encompass various sources such as skin contact with cold objects, exposure to cold liquids (e.g., swimming or cold showers), low ambient temperatures (cold weather, air conditioning), and the consumption of cold foods and beverages [1]. Despite its relatively rare occurrence, with a reported prevalence of 0.07% [2], ColdU predominantly affects young adults, exhibiting a slightly higher prevalence in women. Even though sparse, data on childhood chronic urticaria suggest a prevalence and symptomatology comparable to that observed in adults [3].

The clinical features of ColdU include the rapid onset of pruritic wheals (hives), angioedema (swelling beneath the skin), or a combination of both, typically emerging within minutes after skin contact with cold stimuli and persisting for about an hour [4]. These symptoms are chronic and may recur over a period exceeding six weeks [4]. ColdU signs and symptoms predominantly manifest locally at the specific site or part of the body that came into direct contact with cold stimuli testing [3, 4]. Hives vary in terms of size, shape, number, coalescence, and edema [5], and there are also variations in the severity of itch, burning, and pain for ColdU patients. These urticaria symptoms have a substantial impact on health-related quality of life (HRQoL) [6], with itch being a predominant cause of interference in everyday life [7]. In addition, individuals with ColdU commonly adopt a strategy of symptom management through the avoidance of cold exposure [8], a coping mechanism that, in turn, can significantly disrupt their daily lives [9]. Furthermore, systemic reactions to ColdU, occurring in a considerable percentage of cases (35% to 70%), may include severe manifestations such as anaphylaxis [10, 11]. Children with ColdU are at heightened risk of systemic reactions, including anaphylaxis, underscoring the potentially life-threatening nature of this condition [12, 13]. The overall understanding of ColdU is vital not only for effective clinical management but also for addressing the considerable impact it imposes on the HRQoL of affected individuals.

Although the clinical signs and manifestations of ColdU are documented in literature, there is a noticeable gap in understanding the comprehensive patient experience, spanning signs, symptoms, and impacts described directly from the patients across different age groups. To address this gap, a qualitative interview study was conducted to explore the lived experiences of individuals with ColdU. This approach is in line with the US FDA’s recent guidance on Patient-Focused Drug Development (PFDD) that recommends patients directly report their experience unless they cannot be expected to from reliably self-report. In this case, caregivers/parents could report on what they observe in their child, or what their child has reported to them if they are old enough to verbalise this [14]. The study aimed to identify the most relevant and bothersome signs, symptoms, and impacts of ColdU across adults, adolescents, and children, and to develop the first patient-centric conceptual model depicting the ColdU experience.

## Methods

### Participants and setting

The qualitative study involved semi-structured concept elicitation (CE) interviews conducted in English language with individuals diagnosed with ColdU in the United States (US). The study comprised four distinct cohorts: adults aged 18–80 years, adolescents aged 12–17 years, pediatric patients aged 4–11 years (accompanied by a parent/caregiver), and parents/caregivers of pediatric patients aged 2–3 years. Inclusion criteria stipulated a clinician confirmed diagnosis of ColdU, characterized by the recurrence of itchy wheals and/or angioedema due to cold lasting longer than 6 weeks. Participants were required to have experienced an episode of cold exposure-triggered urticaria within the last 6 months, leading to healthcare provider intervention or the need for prescription medication for symptom control. Participants were excluded from the study if they had a medical, psychological, or cognitive condition that would interfere with their ability to participate in the interviews, had previously participated in a clinical trial for ColdU in the last 6 months or were exposed to/ tested positive for COVID-19 in two weeks prior to the interview.

Recruitment for the study was done through a vendor using their clinician network, social media, and patient association groups. Informed consent/assent was obtained from individuals willing to take part in the study before participation. Approximately 30-minute interviews were conducted via telephone, which were recorded, transcribed verbatim, and anonymized to ensure data integrity and confidentiality.

### Concept elicitation interviews

The CE interviews were conducted between June 2022 and March 2023, and aimed to gain a comprehensive understanding of a patient’s daily life with ColdU. This included the language used by the patients to express each concept, as well as the frequency, severity, and duration of each sign or symptom experienced. Participants were also probed on the triggers and alleviators for each reported concept, as well as the ways in which these signs and symptoms impacted their lives. Additionally, the most bothersome signs and symptoms were elicited from each participant and the reasons for selecting these concepts as most bothersome were explored.

Experienced and trained moderators facilitated the interviews using semi-structured guides tailored to each study cohort. Participants were asked a series of open-ended questions about their experiences with ColdU to encourage spontaneous descriptions of signs, symptoms, and the impacts of living with the condition. Probes were used when specific concepts of interest or topics were not mentioned spontaneously or when the moderators deemed additional information was necessary. In interviews involving adolescent participants, caregivers were allowed to attend but were advised not to contribute to the interviews. For parents/caregivers of patients aged 4–11 years, caregivers provided supportive information, assisting the child if needed. In the case of the caregiver of the patient aged 2–3 years, the parent/caregiver reported the experiences of their child with ColdU, based on their observations or based on what their child described to them through directed open-ended questions.

### Data analysis

A thematic analysis approach [15, 16] was employed to analyze the CE data, and involved identifying patterns in meaning across the interview transcripts to derive themes, using MAXQDA, a qualitative analysis software (2020 [VERBI Software, 2019]). The same team of trained qualitative analysts conducted concept coding for all the transcripts included in this study. They coded the transcripts iteratively, adding, deleting, or merging codes based on their relevance, meaningfulness, or conceptual equivalence identified during the interview analysis. The coders collaborated to reach consensus on updates and changes to the coding framework. To ensure that codes were being applied consistently, dual coding took place for 100% of transcripts to minimize non-systematic bias and inconsistent judgement and to ensure that the data were analyzed in accordance with study objectives. All discrepancies were discussed and reconciled among the qualitative analysts involved in the coding.

To ensure the appropriateness of the sampling and generalizability of the qualitative findings, the principle of saturation was assessed [17]. A stepwise comparison of concepts identified in chronologically ordered groups of interviews was conducted to ensure that any new concept not previously highlighted was duly identified. Conceptual saturation was deemed achieved when no new concepts emerged spontaneously in the final group of interviews.

The insights derived from the interviews helped to inform a comprehensive conceptual model for ColdU, which encapsulates all symptoms, signs, and impacts from the lived experiences of individuals with ColdU.

## Results

### Sample characteristics

A total of 20 participants were recruited and interviewed: adults aged 18–80 years (*n* = 8), adolescents aged 12–17 years (*n* = 5), children aged 4–11 years, along with their caregivers/parents (*n* = 6), and a caregiver of a child aged 2 years (*n* = 1).

Demographic characteristics of adult and adolescent participants revealed a predominant representation of females (85%), individuals of White ethnicity (85%), non-Hispanic or Latino origin (92%), and a majority holding a graduate degree (31%). Among adult participants, ages ranged from 31 to 61 years, with a mean age of 45 years. For adolescent participants, ages ranged from 14 to 16 years, with a mean age of 15 years. In the group of children aged 4–11 years, over half were female (67%), all were of White ethnicity (100%), non-Hispanic or Latino origin (100%), with educational levels ranging from elementary school (83%) to middle school (17%). The mean age in this group ranged from 4 to 11 years, with a mean age of 9 years. The caregiver interviewed was the caregiver of a female patient aged 2 years old (Table 1).

**Table 1:** Demographic characteristics of the cohorts

		Adult and adolescent sample			Children/caregiver sample		
Characteristic		Adults (*n* = 8)	Adolescents (*n* = 5)	Total (*n* = 13)	Children 4–11 years (*n* = 6)	Children 2–3 years* (*n* = 1)	Total (*n* = 7)
Age (years)	Mean (standard deviation)	45 (9)	15 (0.8)	33 (16.1)	9 (2.4)	2 (0)	8 (3.3)
	Min - max	31–61	14–16	14–61	4–11	2–2	2–11
Sex, N (%)	Male	-	2 (40)	2 (15)	2 (33)	-	2 (29)
	Female	8 (100)	3 (60)	11 (85)	4 (67)	1 (100)	5 (71)
Race, N (%)	White	8 (100)	3 (60)	11 (85)	6 (100)	-	6 (86)
	Multi-racial	-	2 (40)	2 (15)	-	1 (100)	1 (14)
Ethnicity, N (%)	Non-Hispanic or Latino	-	-	-	6 (100)	1 (100)	7 (100)
	Hispanic or Latino	-	1 (20)	1 (8)	-	-	-
Highest level of education, N (%)	Elementary school		-	-	5 (83)	-	5 (71)
	Middle school	-	1 (20)	1 (8)	1 (17)	-	1 (14)
	High school	-	4 (80)	4 (31)	-	-	-
	Some college	1 (13)	-	1 (8)	-	-	-
	Associate degree	2 (25)	-	2 (15)	-	-	-
	Bachelor’s degree	1 (13)	-	1 (8)	-	-	-
	Graduate degree	4 (50)	-	4 (31)	-	-	-
	Missing	-	-	-	-	-	1 (14)
Location, N (%)	Maryland	-	-	-	2 (33)	-	2 (29)
	Florida	-	-	-	-	1 (100)	1 (14)
	Michigan	2 (25)	-	2 (15)	-	-	-
	Kentucky	-	1 (20)	1 (8)	-	-	-
	California	-	1 (20)	1 (8)	-	-	-
	Arkansas	1 (12)	-	1 (8)	-	-	-
	New York	1 (12)	-	1 (8)	-	-	-
	New Jersey	1 (12)	-	1 (8)	-	-	-
	North Carolina	1 (12)	-	1 (8)	-	-	-
	Colorado	-	1 (20)	1 (8)	-	-	-
	Missing	2 (25)	2 (40)	4 (31)	4 (67)	-	4 (57)

– no participant with this characteristic included; N = total number of participants; * Only caregiver was interviewed

### Concept elicitation interview content analysis

#### Adult and adolescent participants

A total of 22 distinct symptoms/signs were reported by the 13 participants, revealing very similar experiences between adults and adolescents (Fig. 1a). The experience of ColdU described by the adult (*n* = 8) and adolescent (*n* = 5) participants revealed hives and itch to be the two most relevant symptoms/signs of the disease, reported by all 13 participants.

**Fig. 1a:**
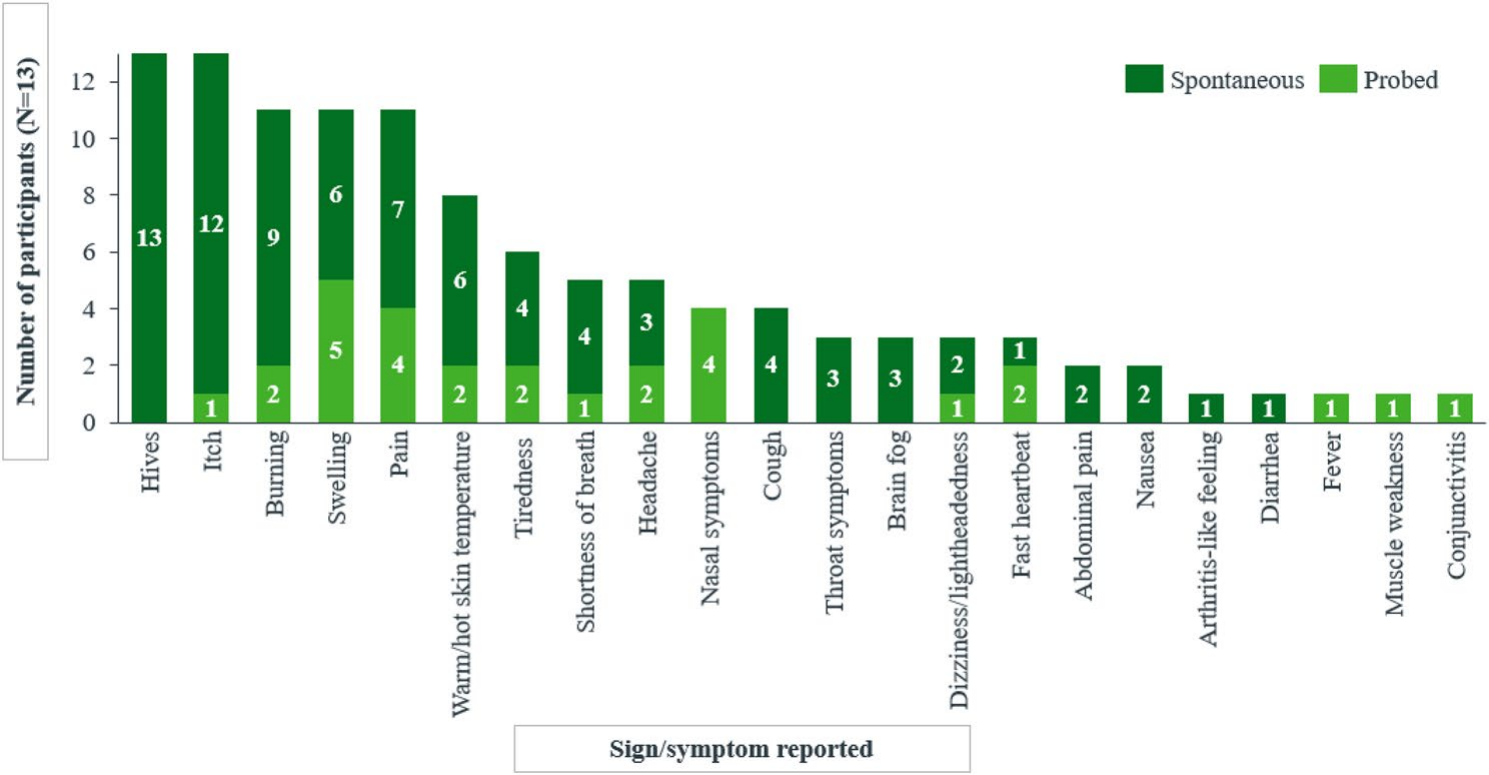
Number of adult and adolescent participants reporting each symptom/sign spontaneously or probed

**Fig. 1b:**
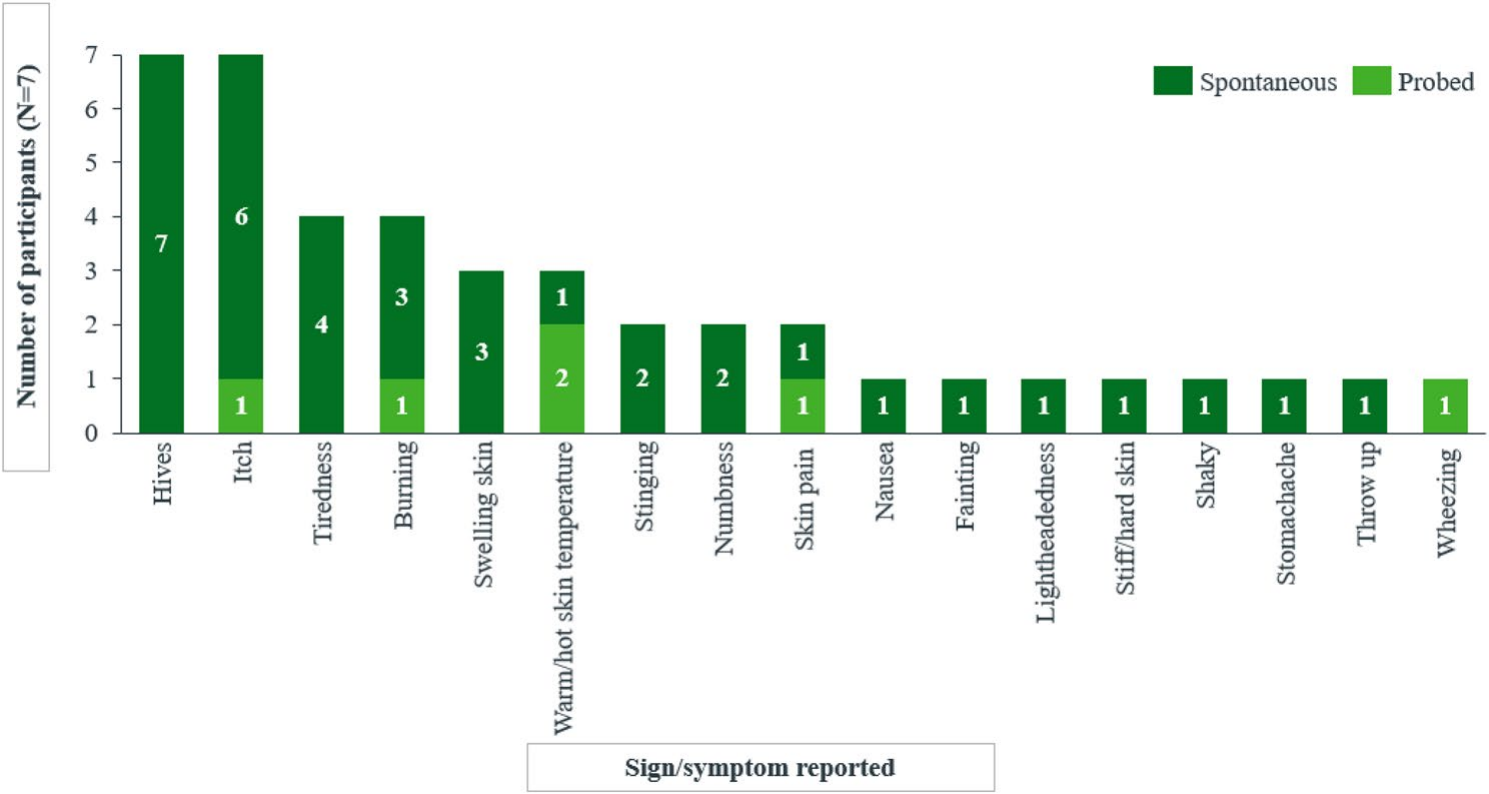
Number of child and caregiver participants reporting each symptom/sign spontaneously or probed

*I’ll usually start to see some redness and then I just get…I guess the medical term*, ***these wheals that pop up***. (P-02-44-F)

*It slowly progresses to being* ***itchy*** *…it feels like a mix between mosquito bites and like a* ***poison ivy rash****…* (P-01-31-F)

In addition, most of the participants reported experiencing burning (*n* = 11/13), swelling (*n* = 11/13), pain (*n* = 11/13), and warm/hot skin temperature (*n* = 8/13).

*…it’s* ***like pins and needles***… *when you try to…* ***close your hands and your fingers are all burning***. (P-11-14-M)

*My hands were getting* ***swollen…my knuckles were huge***,*** and everything was itchy*** (P-11-14-M)

*…But when I’m* ***drinking a Diet Coke***, *I’ll feel my lips tingling and I know they’re probably swelling just a little bit.* (P-03-61-F)

*… it is* ***painful*** *when the* ***hives start to form***, *and I start to get those* ***little bumps****…* (P-01-31-F)

Participants explained that hives were triggered by contact with or exposure to cold stimuli such as cold water/weather and air conditioning, and that those were accompanied by itch, swelling and sensations of burning, pain, and warm/hot skin. Specifically, hives were consistently described as red reactions on the skin, varying in size and appearing early (within minutes) when there was exposure to cold, and preceding other symptoms including itch and pain. Participants also reported experiencing these hives more frequently during the winter months when exposure to cold weather was more frequent. As ways of alleviating these reactions most participants reported warming oneself up (n = 11) and distancing oneself from the cold trigger (n = 5). Alleviation could be as quick as within 5 minutes (n = 2) or as long as up to 90 minutes (n = 2). Most patients described their hives as itchy. They also reported swelling occurring concurrently with hives, and, in some cases, could affect entire body parts, such as hands and fingers. Swelling was often reported associated with burning and pain around the itchy wheals, and participants indicated it manifested instantly to within minutes of cold exposure. These concurrent symptoms/signs of itch, swelling, burning, pain and warm/hot skin were reported to be alleviated within minutes/hours after distancing from the cold trigger leading to the onset of symptoms. Lastly, pain was commonly described using terms such as ‘pain’ or ‘hurt,’ and noted that it often occurred after the development of hives and swelling. Amongst other signs and symptoms, patients reported experiencing burning sensation, that they described ‘like fire’, ‘like insect bites/sting,’ or ‘like pins and needles.’ Importantly, patients conveyed that while burning was a distinct sensation, it was closely related to, yet different from, the experience of pain. In addition, patients mentioned experiencing warm/hot skin temperature, using terms centered around heat, such as ‘hot,’ ‘warm,’ and ‘heat.’ This sensation was most frequently reported on the hands, although areas of the head, including the ears and cheeks, were also reportedly affected (Table 2).

**Table 2:** Participant quotes describing key signs and symptoms elicited from adults and adolescent patients

Symptom	Illustrative quotes
Hives	• *“* ***I get the hives all over me****… It gets* ***really red****… I get hives and I get* ***little welts*** *on it*,* welts on them.”* (P-09-16-F) • *“When I first got diagnosed*,* I had a 10-week urticaria outbreak. It was the dead of winter here…* ***my entire body was itchy for 10 weeks****…* ***It’s usually centered around where the hives are***.*”* (P-02-44-F) • [When asked about most bothersome symptoms] “***The blotchiness or hives****… Because* ***then I know that it’s a more severe reaction***, *and it’s going to take more time to subside*.” (P-12-37-F)
Itch	• [When asked about most bothersome symptoms] *“Definitely* ***the itchiness****… It’s just* ***so incredibly uncomfortable*** *when it’s bad. Because you can’t itch it because you could make your skin get worse with it. It’s just like the most frustrating thing because it’s super itchy*,* but if you itch it*,* you could accidentally hurt yourself.”* (P-11-14-M)
Swelling	• *“I was actually genuinely really worried because my fingers they were* ***swelling*** *bad. I felt like my* ***fingers were about to explode***.*”* (P-08-15-M) • *“…all parts of my body*,* you know*,* not like just isolated to my hands or to my cheeks or ears. It would be* ***like my entire body would breakout****…” (P-04-53-F)*
Pain	• “*My hands get swollen*,* and then* ***they really hurt***.” (P-07-52-F). • [When asked about most bothersome symptoms] “The pain… It’s extreme pain and it’s uncomfortable. I could look blotchy, I don’t care, but if that pain went away, I would be very happy.” (P-07-52-F)
Burning	• *“Sometimes with the hives and the rashes*,* they burn.* ***Like it doesn’t really hurt***,*** it just burns****… it is a little hard to put into words*,* but I remember it’s kind of like when your hand falls asleep*,* for example*,* and it’s like pins and needles.”* (P-11-14-M)
Warm/hot skin temperature	• *“****The feeling hot on the palms of my hands and my earlobes***, *and it’s usually after I’ve been in contact with something cold*,* and the burning starts then it just starts feeling hot.* ***It actually feels warm to the touch***.*”* (P-10-42-F)

Legend for participant IDs included alongside the quotes: First letter indicates that the interview participant was a patient (P). The next two digits indicated the participant number, which were chronologically assigned by the order in which participants were recruited. The following two digits refer to the age of the patient. The final letter indicates whether the patient was male (M) or female (F)

Reports of the most bothersome symptoms/signs varied, with nine different symptoms identified as among the most bothersome. Notably, itch (*n* = 3), hives (*n* = 3), and pain (*n* = 3) were consistently reported as the most bothersome by both adults and adolescents (*n* = 13/13) due to being persistent or uncomfortable (Table 2).

A total of 32 impacts across seven HRQoL domains were identified. All adult and adolescent participants reported impacts on activities of daily living (n = 13/13). A majority mentioned that their hobbies (n = 12/13) and clothing (n = 11/13) were affected by their condition.

*I was told also not to* ***jump in water like in a lake***. *Just to slowly go in because I’ll go into shock. We have property up north and we would go* ***swimming****…* ***I come out I’ll have welts all over my body***.* (P-07-52-F)*

***I play the flute****—I’m in marching band—and* ***it can be really hard to keep my fingers moving when they’re really cold and itchy***, *and keep playing because I just… I need to scratch them. (P-09-16-F)*

*I* ***dress different than most people****… If I’m outside*,* other people would be wearing jeans.* ***I probably would be wearing ski pants***. (P-03-61-F)

Emotional impacts, including frustration, were reported by most participants (n = 12/13), along with physical impacts on activities like sports and exercise (n = 11/13), social impacts related to participation in social activities (n = 10/13), and impacts on work/school activities (n = 9/13), most of which were reported spontaneously.

*…because of wintertime if the grandkids are going downhill skiing*,* and I’ve been doing that with them before*,* and* ***now I can’t go along with them***, *so just different situations like that that become* ***frustrating***.* (P-04-53-F)*

***The depression of not being able to do things like everybody else does.*** *They don’t have to think twice. They don’t have to pack extra clothes in the car. They don’t have to think about all these things. They don’t have to*,* yeah*,* they just…most people don’t understand how much it* ***impacts your life*** *and how many things around you are actually cold and dangerous.* (P-10-42-F)

Less frequently reported impact domains included sleep (*n* = 5/13) and financial impacts (*n* = 1/13) (Fig. 2a).

**Fig. 2a:**
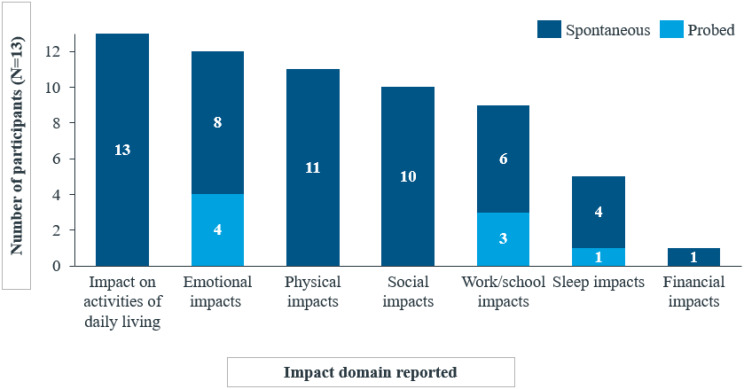
Overview of impact domains reported by adult and adolescent participants spontaneously or probed

**Fig. 2b:**
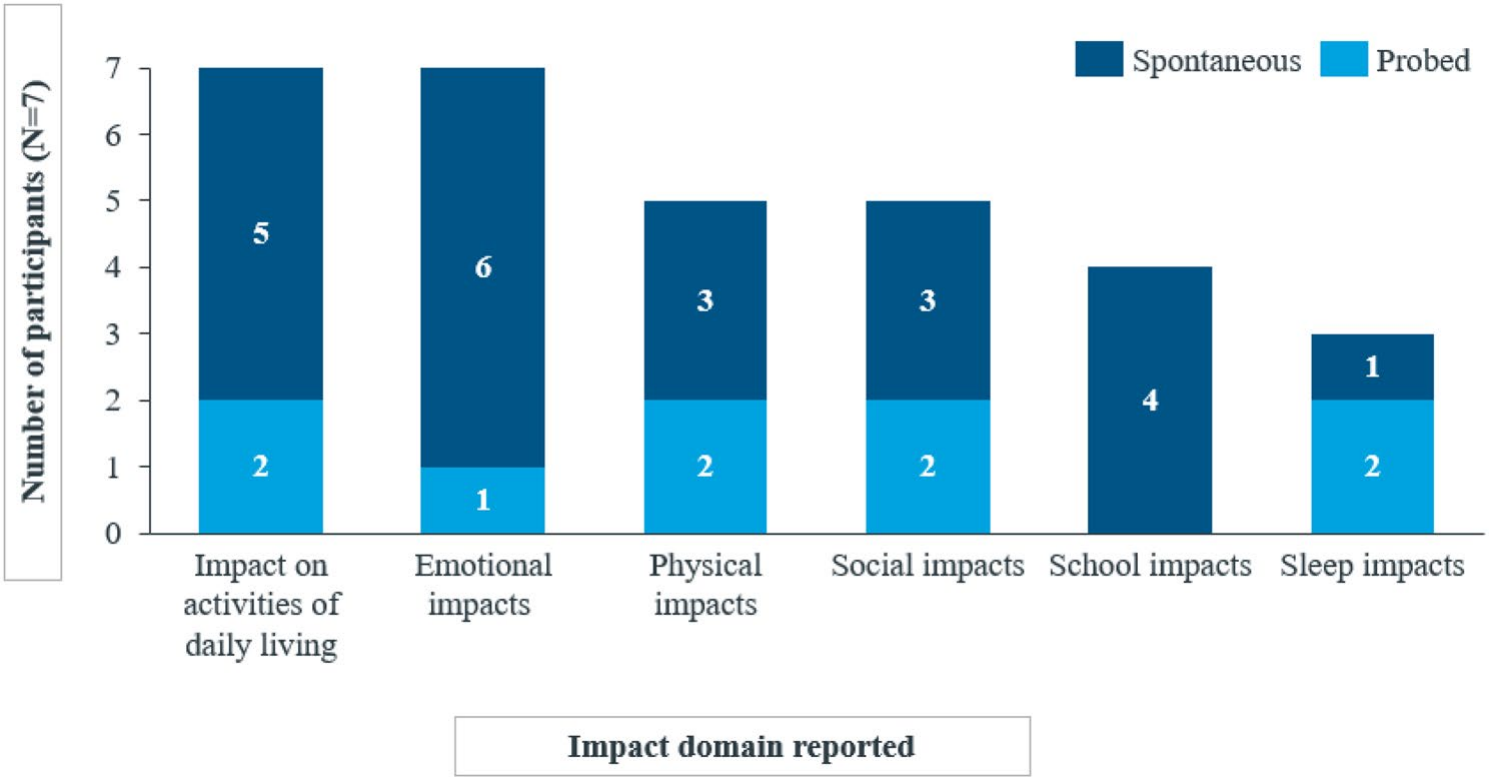
Overview of impact domains reported by child and caregiver participants spontaneously or probed

The experiences with ColdU on HRQoL were similar between adults and adolescents, with differences typically related to age (e.g., work/school impacts) and reported by only a small number of participants. Work impacts were reported exclusively by adults (*n* = 6), while school impacts were reported only by adolescents (*n* = 3). Sleep impacts were reported by one adolescent compared to four adults. Financial impacts were exclusively reported by adults, with only one participant mentioning these impacts (Fig. 3a).

**Fig. 3a:**
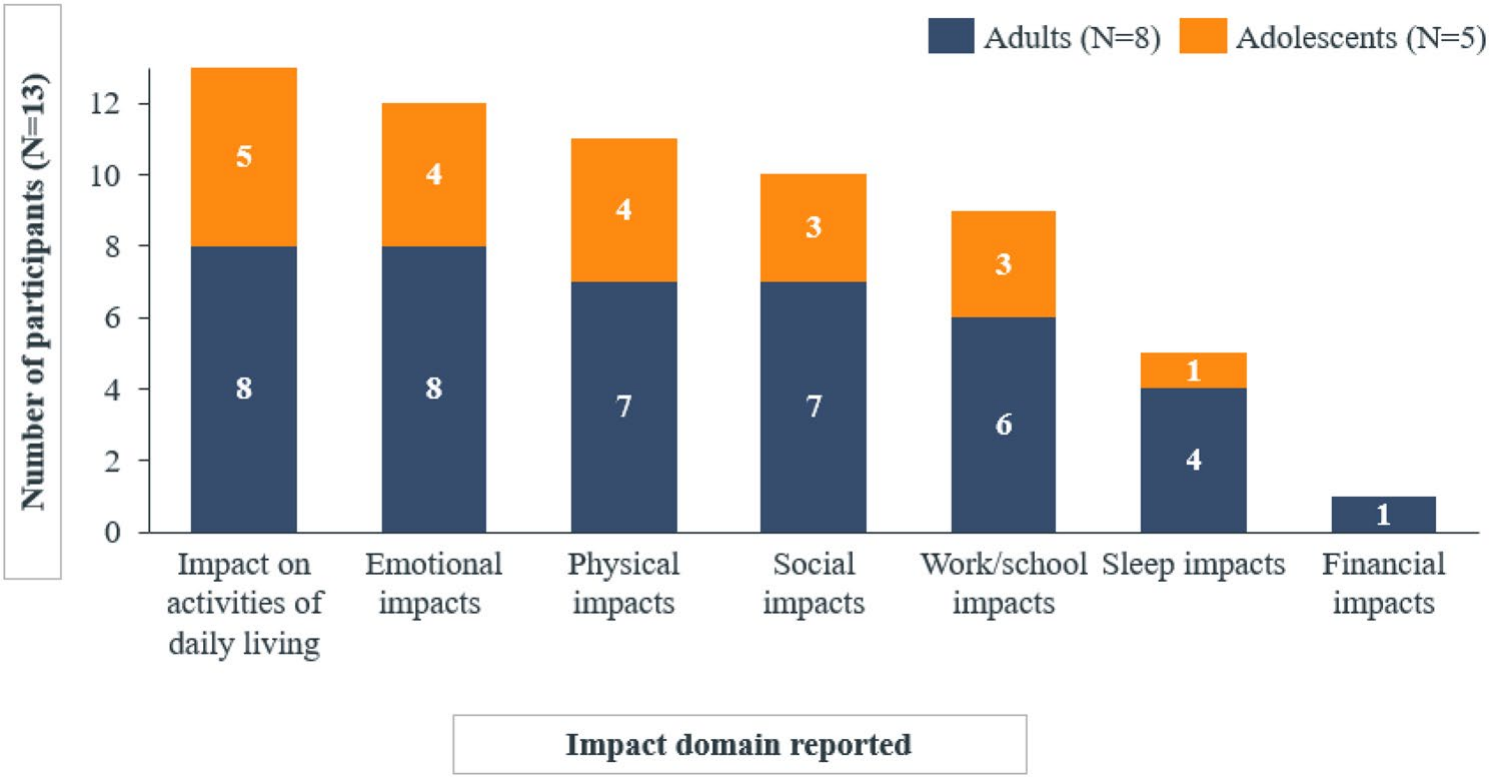
Comparison of impact domains reported by adult and adolescent participants

**Fig. 3b:**
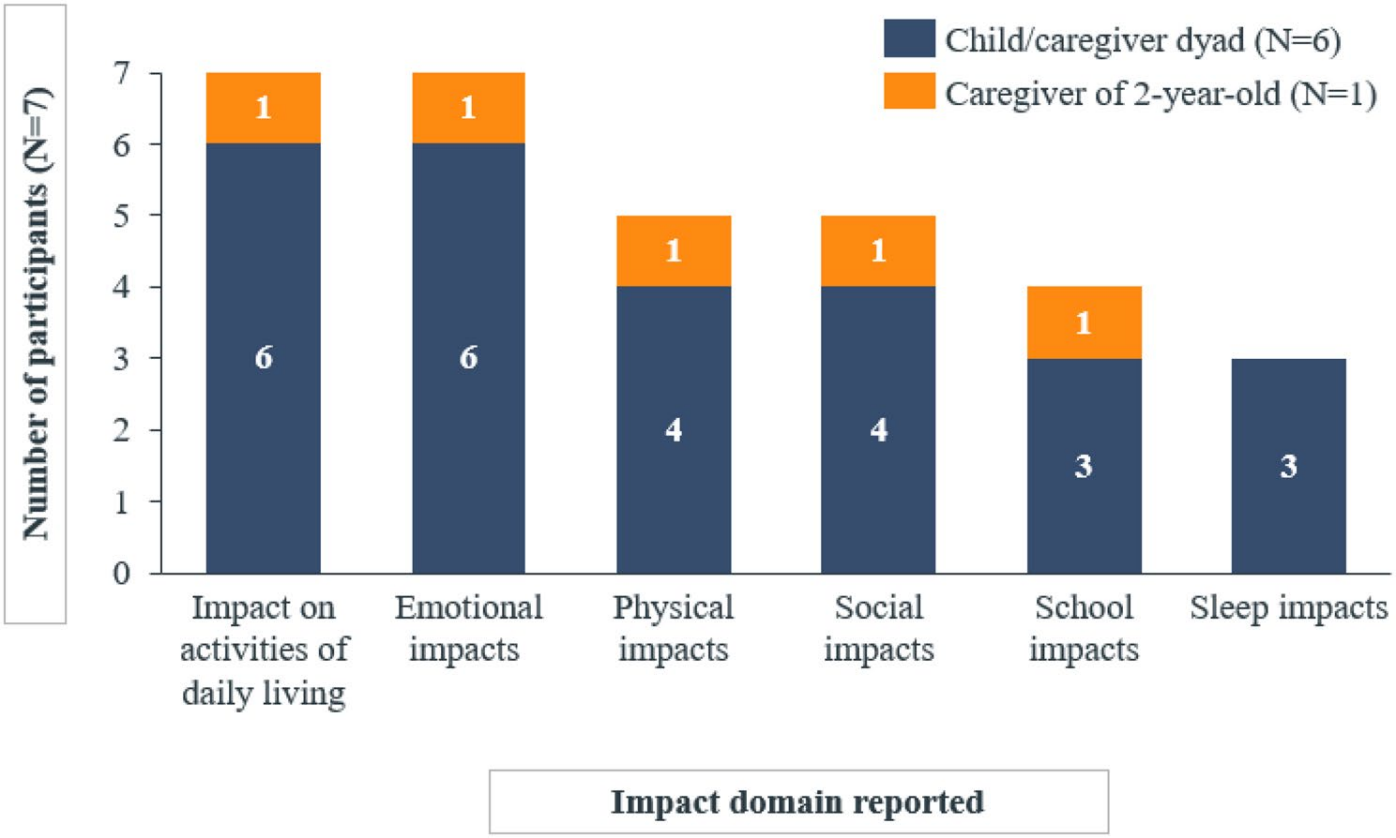
Comparison of impact domains reported by child/caregiver dyads and solo caregiver participants

Saturation analysis for the reported symptom/sign and impact concepts for the adult and adolescent sample are shown in Tables 3 and 4, respectively. The saturation analysis indicated that most of the symptom/sign concepts were reported spontaneously for the first time in the first group of three interviews (Table 3). Only two concepts (nausea and diarrhea) emerged for the first time in the final set of interviews, specifically during the 11th interview. However, these two concepts were probed in the preceding 10 interviews but were not experienced by any of these participants. Overall, nausea was experienced by only two of the 13 participants, and diarrhea by just one participant, indicating that these symptoms are not common in ColdU. Similarly, impact concepts within all HRQoL domains were reported spontaneously for the first time in the first group of three interviews (Table 4).

**Table 3:**
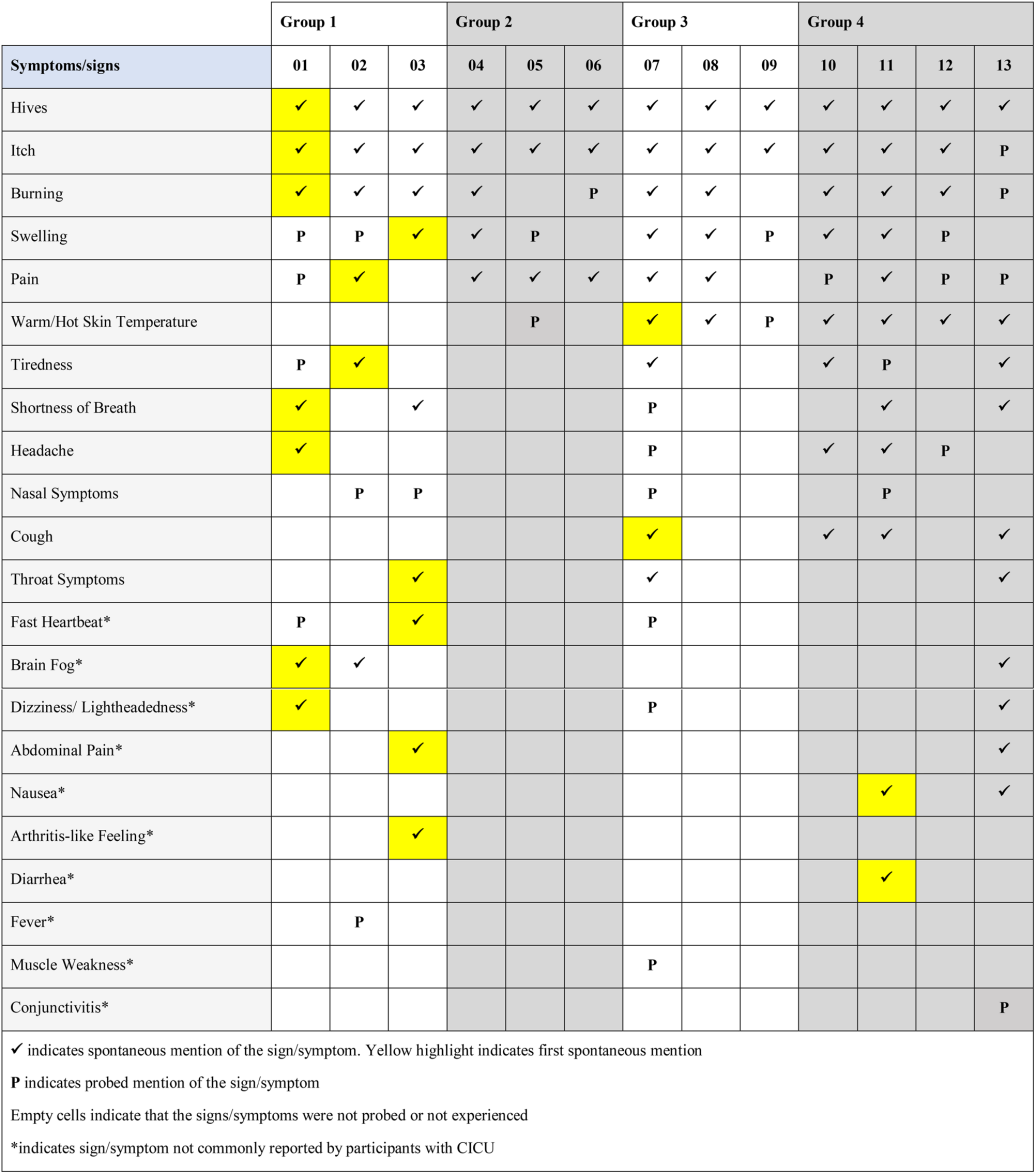
Saturation analysis for symptom/sign concepts reported by adults (*n* = 8) and adolescents between 12–17 years (*n* = 5)

**Table 4:**
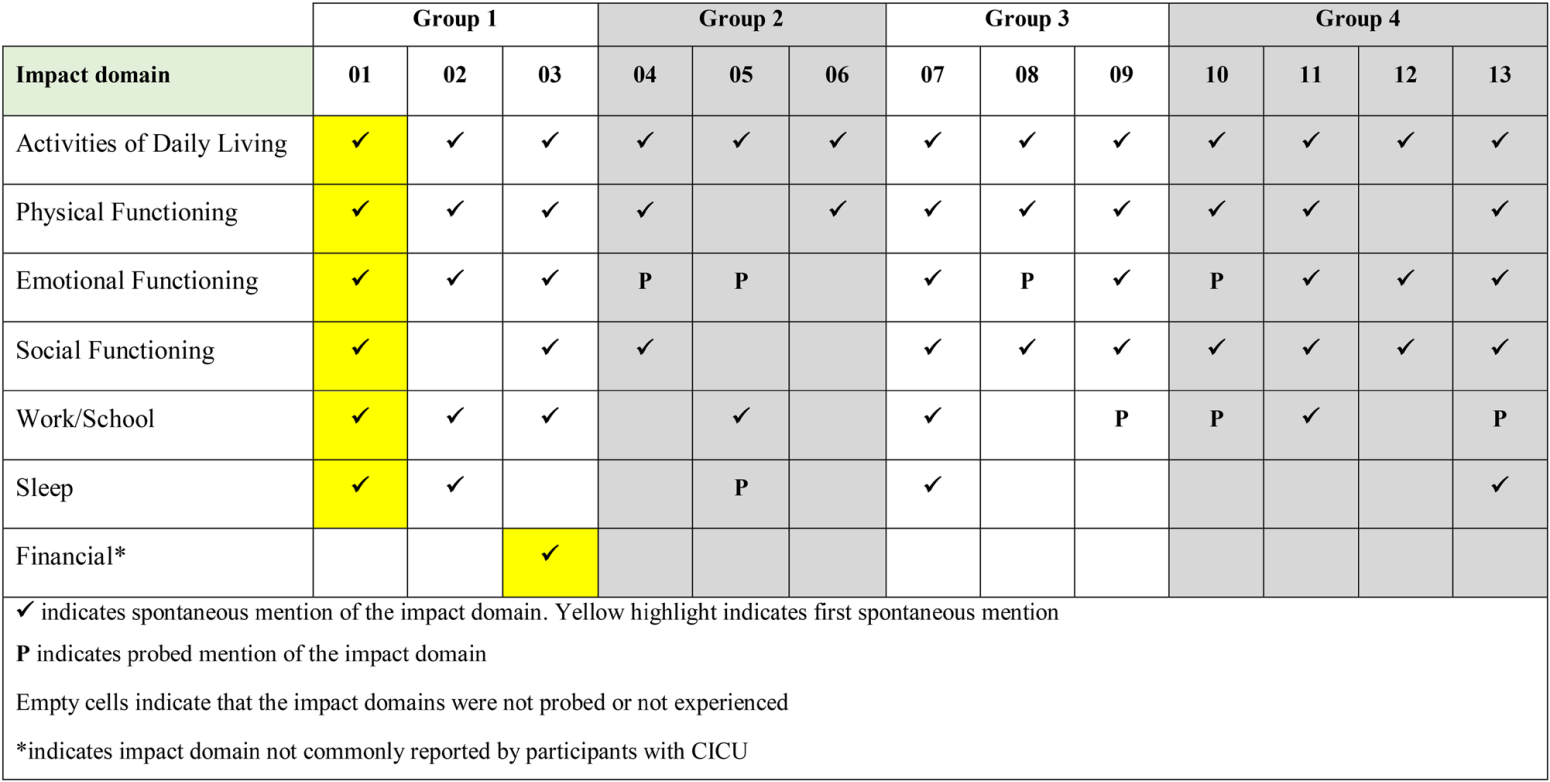
Saturation analysis for impact domains reported by adults (*n* = 8) and adolescents between 12–17 years (*n* = 5)

#### Children participants with ColdU

In the case of 7 children and/or their caregivers, a total of 17 ColdU-related symptoms/signs were identified. Like with the adults and adolescents, interviews with children and/or their caregivers suggested hives and itch to be the defining characteristics of ColdU, being reported by all participants (*n* = 7/7).

*A lot of the times when I’m cold*, ***I get red spots***. (DY-01-10-F-P)

*Like mixed together*,* it’s* ***like itchy and stingy***. (DY-04-09-F-P)

*…****normally I’m itchy with the hives***. (DY-04-09-F-P)

Other frequently reported concepts included tiredness (4/7), burning (n = 4/7), swelling (n = 3/7), and warm/hot skin temperature (n = 3/7) (Fig. 1b).

*I feel that* ***where the cold is the most***, *which is mostly on* ***my thighs***, *and then on* ***my arms*** *too*,* and* ***my stomach****… When I come inside from the cold*, ***my ears they burn and then my cheeks do too****…* ***My hands sometimes***.* (DY-01-10-F-P)*

*It’s* ***the air*** *and then if* ***the snow*** *touches her*,* it definitely…she typically* ***swells*** *there.* (DY-05-11-F-CG)

*I felt the hives and I felt that* ***the hives was hot***. (DY-04-09-F-P)

Experiences of symptoms and signs were generally similar between children aged 4–11 years and those of the 2-year-old child. For several symptoms, including burning and pain the child told their caregiver about the symptom and this was reported during the dyad interviews.

Hives were typically described as a general redness of the skin, with a few participants also using terms such as ‘bumps’ and ‘welts,’ among others, and varying in number and in size. Specifically, the caregivers reported seeing hives that looked ‘red,’ ‘swollen,’ and ‘blotchy.’ In parallel, some of the children provided vivid descriptions of their itch experience associated with these hives, characterizing it as ‘tingly’ or feeling ‘like a bug bite’ or ‘like ants crawling on you’. Itchy hives were reported to appear instantly after cold exposure and require medication and/or warming up (with additional clothing, warm baths) to be alleviated. While most participants reported that itchy hives could be alleviated within minutes of getting away from the cold exposure, one caregiver of a child reported that it could take until the next day for the redness to disappear. Child patients also described experiencing burning sensation upon cold exposure and discussed having swelling skin that some children described as ‘puffy,‘; the infant’s caregiver referred to it as a ‘bad reaction to a bug bite’. Amongst other signs and symptoms, most children and the infant’s caregiver mentioned a skin feeling warm or hot, most frequently experienced in areas directly exposed to cold stimuli, where hives were present, and specifically on the ears, hands, and arms (Table 5).

**Table 5:** Participant quotes describing key signs and symptoms elicited from child patients and /or their caregivers

Symptom	Illustrative quotes
Hives	• *“They were like* ***different sizes of bumps***.*”* (DY-04-09-F-P) • *“I get these* ***tiny bumps***.*”* (DY-01-10-F-P) • [When asked about most bothersome symptoms] *“Probably* ***the hives***, *honestly*,* because* ***they happen a lot more*** *and* ***they’re just really annoying***, *I guess.”* (DY-04-09-F-P)
Itch	• *“****My hands get tingly****…It feels kind of* ***like ants are crawling all over you***.*”* (DY-02-11-F) • [When asked about most bothersome symptoms] *“Now that she’s verbalizing that* ***they’re itchy***, *I think she’s aware of them and she is experiencing discomfort and is letting me know that she is experiencing discomfort.”* (CG-01-02-F)
Tiredness	• *“I almost feel like he becomes a little on the* ***lethargic*** *side.” (DY-03-04-M-CG)* • *“…* ***she was exhausted*** *when she got out.” (DY-02-11-F-CG)* • *“…****Fatigue***, *most definitely …* ***but my whole body feels heavier to lift up.****”* (DY-02-11-F-P)
Swelling	• *“The welts were first*,* and like I said*,* they started small*,* they got bigger*,* they got more widespread. As that was happening*, ***I’m watching her swell***.*”* (DY-05-11-F-CG) • *“They get very swollen*, ***very puffy***.*”* (DY-02-11-F-P) • *“It’s swollen localized to where the cold was*,* and it looks* ***like somebody had a bad reaction to like a bite***, *a bug bite or an ant bite or something like that.”* (CG-01-02-F)
Burning	• *“****When the leggings got rained on***,*** they got cold and then the cold got on my legs and then it started burning…*** *I feel that* ***where the cold is the most***, *which is mostly on* ***my thighs***, *and then on* ***my arms*** *too*,* and* ***my stomach****… When I come inside from the cold*, ***my ears they burn and then my cheeks do too****…* ***My hands sometimes…*** *I get it* ***mostly in winter or fall***, *whenever it’s cold*,* but definitely not summer unless I’m swimming in lakes. I definitely do get it a lot*.*”* (DY-01-10-F)
Warm/hot skin temperature	• *“Her skin is* ***warm to the touch*** *where that cold was.”* (CG-01-02-F) • *“Well*,* sometimes* ***my skin just feels like really hot***, *and I can’t control it… My* ***hands and my arms*** *mainly.”* (DY-05-11-F-P)

Legend for participant IDs included alongside the quotes: First letter indicates that the interview type was a dyad (DY) or a caregiver only (CG). The next two digits indicates the participant number, which were chronologically assigned by the order in which participants were recruited into dyad or solo caregiver interviews. The following two digits refers to the age of the patient. The following letter indicates whether the patient was male (M) or female (F). If a dyad interview, the final letter indicates whether the quote was said by the caregiver (CG) or the child/patient (P)

Lastly, the infant’s caregiver reported their child being ‘lethargic’ and ‘being exhausted’, while children patients reported feeling ‘fatigue’ and ‘feeling heavy in the body’.

*She was just* ***splashing in the water***, *and within a matter of 5 minutes she was covered head to toe in hives and started becoming very lethargic.* (CG-01-02-F)

*“When I’m cold*,* I feel definitely tired. I definitely feel like*,* I don’t know how to really say this*, ***but my whole body feels heavier to lift up.****”* (DY-02-11-F-P). Four different symptoms were identified as being among the most bothersome, with hives (*n* = 3) and itch (*n* = 2) consistently reported as the most bothersome by children and the 2-year-old’s caregiver, because of the discomfort due to these symptoms/signs and the frequency at which they occurred. One participant (*n* = 1) reported stinging as most bothersome symptom as it was a newly developed symptom. Another participant (*n* = 1) reported fatigue as most bothersome due to its severity (Table 5).

Nineteen impacts across six HRQoL domains were identified for children and caregiver participants. All children and caregiver participants reported impacts on activities of daily living (n = 7/7), including hobbies (n = 7/7), preparedness (carrying items of clothing everywhere they go) (n = 5/7), and clothing (n = 5/7).

*I was not able to* ***go swimming outside*** *at all. (DY-02-11-F)*

*It’s* ***a pain in the butt if I ever want to go into the snow***. *My mom makes me wear a t-shirt and then sweater and then a coat.* (DY-02-11-F)

*Like in the* ***wintertime***,*** he truly like can’t go in the snow walking*** *to the car and in the house because when the snow touches him*,* he screams like it’s acid. (DY-03-04-M-CG)*

*“I also at the start of the year*,* I have to bring a set of items for the nurse to just keep. There’s the* ***gloves***,*** the emergency gloves***,*** the emergency blanket***,*** the EpiPen and the Benadryl***,*** hand warmers***.*”* (DY-02-11-F). Most participants experienced emotional impacts, including frustration (*n* = 7/7), physical impacts such as sports and exercise (*n* = 5/7), impacts on social activities (*n* = 5/7), impacts on school activities and preschool lives (*n* = 4/7), and poor quality sleep (*n* = 3/7) (Fig. 2b), with the majority being spontaneous reports, highlighting their salience to the patients’ experience of ColdU.

*Yeah. Also*,* I* ***get very worried***. *So the other day at recess*,* I put my stuff…I hit myself with a basketball and I went to the nurse to get an ice pack.* ***I took my medicine that day***,*** but I was a little worried that I would get hives***,*** but I didn’t***. (DY-04-09-F)

*I feel that I can* ***miss out on so much winter activities or water activities*** *with what I have.* (DY-01-10-F)

Few reports of social impact were spontaneous, and sleep impacts such poor sleep quality and increased sleep due to tiredness were reported by children only (Fig. 3b).

Given that only a limited number of interviews (*n* = 7) were conducted with children and/or their caregivers, due to recruitment challenges during the study, saturation analysis was not conducted for these groups.

### Development of the disease conceptual model

Based on the evidence derived from the qualitative interviews, consolidated conceptual models were developed for the sign, symptom and impact experiences of adults (18 years and above) and adolescents (12–17 years) with ColdU (Fig. 4a), as well as for children aged 4–11 years and caregivers of children aged 2–3 years with ColdU (Fig. 4b). Together these models illustrate the multifaceted experience of living with ColdU, depicting the signs, symptoms, and impacts.

**Fig. 4a:**
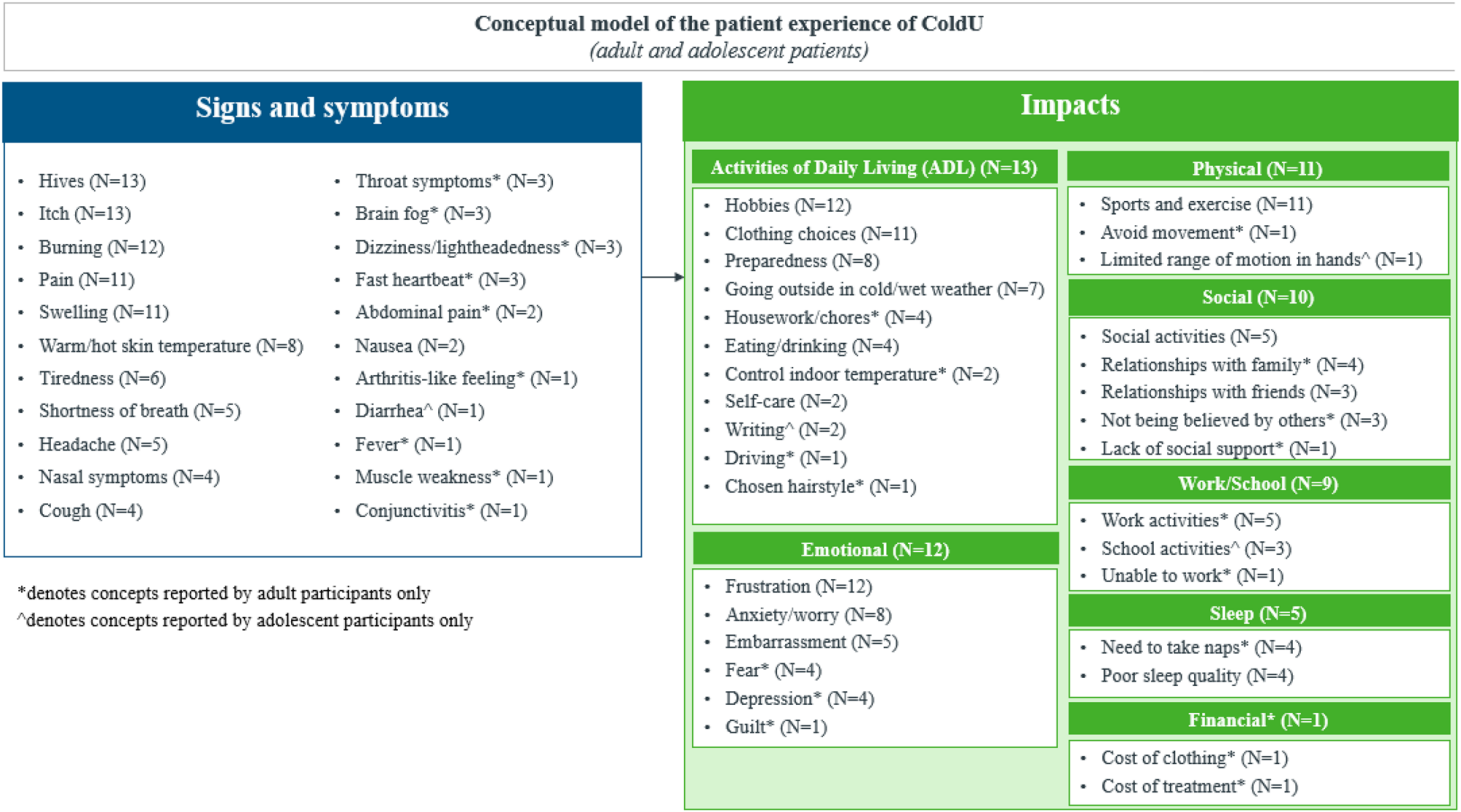
Conceptual model of ColdU signs, symptoms and impacts for adults (*n* = 8) and adolescents between 12–17 years (*n* = 5)

**Fig. 4b:**
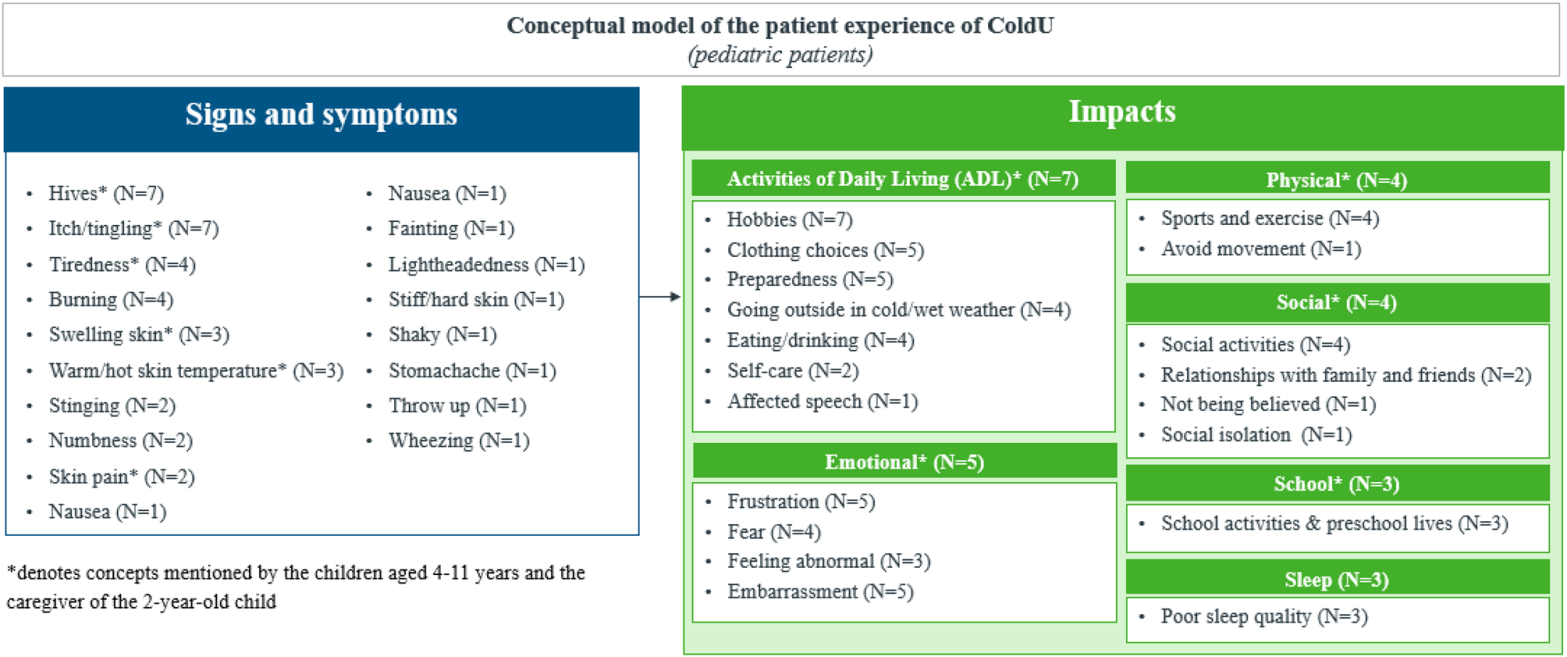
Conceptual model of ColdU signs, symptoms and impacts for children aged 4–11 years (*n* = 6) and the caregiver of a 2-year-old child (*n* = 1)

## Discussion

**Fig. 5:**
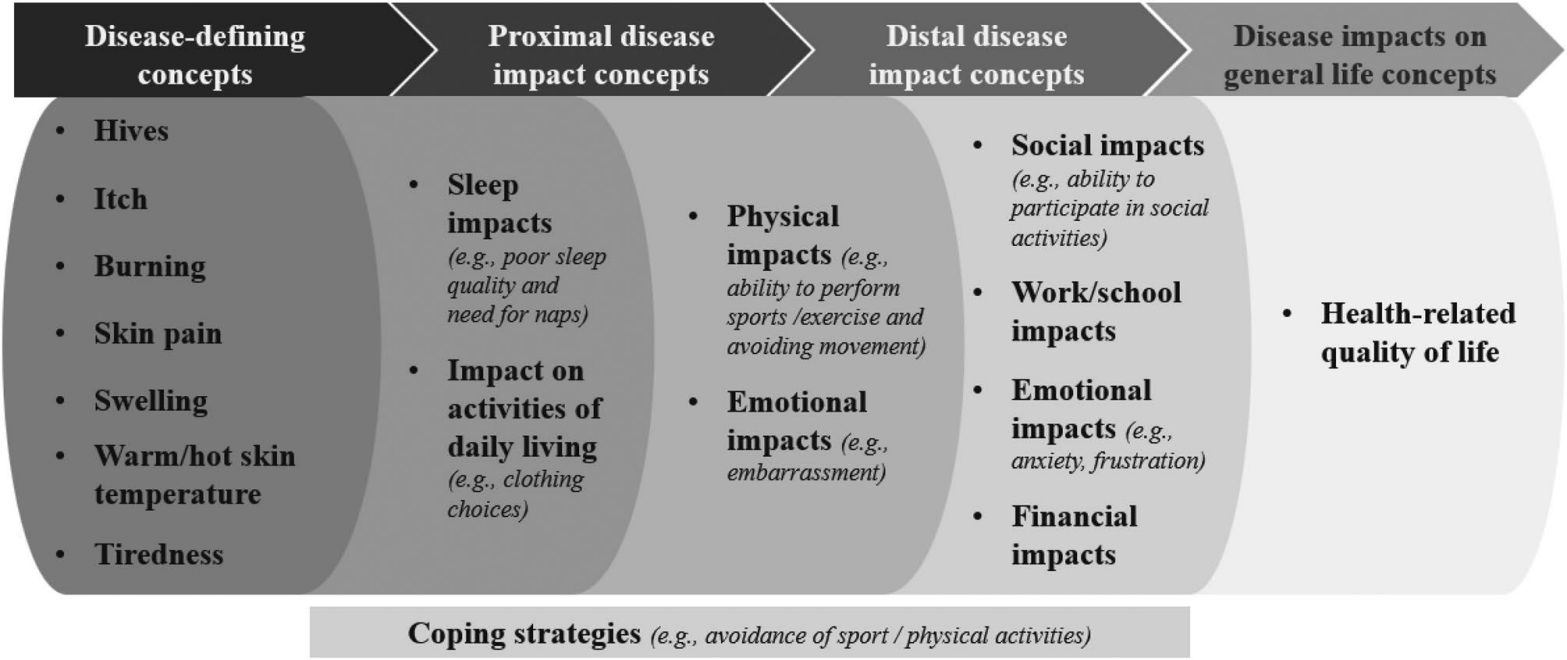
Patient experience of ColdU disease [21]

Published evidence on the lived experience of ColdU is scarce or virtually nonexistent. Existing information primarily relies on clinical manifestations and reporting [18], clinical guidelines [3], and a limited number of national surveys and patients’ testimonials, predominantly in adults [6, 19, 20]. Recognizing this gap, our qualitative interview study was designed to gain a deep understanding of the patient’s experience with ColdU directly from the individuals affected. To our knowledge, this study represents the first of its kind to report the experiences of signs, symptoms, and impacts in adults, adolescents, and children (through their parent/caregiver) with ColdU. ColdU has a detrimental impact on patients’ HRQoL, primarily through hives and itching, two key disease-defining signs and symptoms considered as most bothersome aspects of living with ColdU. In addition, patients also experience burning, swelling, skin pain, warm skin, headaches, breathing difficulties, and fatigue. These symptoms impact daily activities, clothing choices, hobbies, sleep quality, and exercise. Embarrassment from hives or swelling limits social life, causing frustration and anxiety. Professional and school activities are also affected, with financial impacts from treatment and clothing costs. Patients adopt coping strategies to avoid cold exposure, disrupting their daily lives (Fig. 5) [21].

Findings were consistent across age groups, with adults reporting work-related impacts and adolescents reporting school-related impacts. Children’s lives were similarly impacted in areas like daily activities, emotional well-being, social life, and school activities. Unique to children were issues like poor sleep quality, extra preparation time, and feeling abnormal. The findings highlighted the broad impact of ColdU on children, providing valuable insights into their experiences.

Saturation analysis for the combined sample of adult and adolescent participants indicated that saturation was maintained and consequently there is a low likelihood of identifying new concepts with further interviews in these age groups. For the younger populations, saturation was not conducted due to the small number of children and/or their caregivers included in this study. As such, the model derived from these data may be incomplete, however, they provide first and valuable insights about the experience of children patients. These findings can serve as a starting point in the characterization of the detrimental impact of this disease on the HRQoL of younger ColdU populations.

This study significantly contributes valuable and novel perspectives, enhancing our understanding of the signs, symptoms, and day-to-day impacts of ColdU on patients across different age groups. Due to the recruitment challenges it was not possible to actively recruit an ethnically diverse sample (two non-white and one Hispanic participant interviewed). Also, the recruited sample included only a small proportion of males (*n* = 4/ 20), although this might be due to ColdU being more frequently diagnosed in females. Despite the sample diversity limitations, the results from the CE interviews shed light on hives and itch being the core symptoms, and on the debilitating nature of ColdU and its substantial impact on the HRQoL of adult, adolescent and children patients. The interviews underscored the pervasive effects on activities of daily living and emotional well-being, and aligned with existing literature that emphasizes hives and itch as cardinal symptoms [6].

Our study also has several limitations. First, some of our data is presented in a numerical way (for instance, the numbers of participants experiencing specific symptoms). Although this way of presenting is generally preferred by regulators, it does not fully do justice to the richness and complexity of qualitative data and deviates from the traditional principles of qualitative research. Relatedly, the relationships between the experienced concepts were not explicitly explored via moderator probing during the interviews, since the objective was to elicit relevant concepts and explore impact and bothersomeness. Although spontaneously mentioned relationships were analyzed to develop the proximal/distal model in Fig. 5, it would be worthwhile to explicitly study these relationships between concepts in more detail in future qualitative studies. This could contribute to an even better understanding of the patients’ disease experience.

Also, our sample did not include patients with experience of severe reaction to cold. Indeed, even though anaphylaxis or difficulty breathing due to swelling of the tongue or throat could occur in ColdU [3], such severe reactions were not mentioned by the patients interviewed in this study. However, our participants described how the swelling causes impact on their daily life (like eating, writing), physical impacts (on mobility) and emotional impacts (like embarrassment due to the swelling).

Lastly, the challenges faced during recruitment and resulting in the single pediatric participant/caregiver means that the conceptual model for this age group can only be considered preliminary. Further qualitative interviews are needed to refine this pediatric conceptual model. Similarly, due to recruitment challenges, no participants aged between 17 and 30 years old were included in this study. The signs, symptoms and impacts of ColdU as experienced by the participants interviewed may, therefore, differ from experiences of younger adult patients with ColdU.

## Conclusion

The conceptual models developed in this study are the first in-depth characterization of the patient experience with ColdU, offering valuable insights for developing patient-reported measurement strategies and designing future clinical programs. This research fills a crucial gap in understanding ColdU and aims to improve outcomes and decision-making for patients in real-world and clinical settings [22]. The study’s findings lay a strong foundation for addressing the complex challenges of ColdU and enhancing patients’ well-being.

## Data Availability

The semi-structured interview guides and transcripts used and/or analyzed during the current study are available from the corresponding author on reasonable request.

## Abbreviations

ColdIU: Chronic inducible cold urticaria
HRQoL: Health-related quality of life
CE: Concept Elicitation
PFDD: Patient-Focused Drug Development
IRB: Independent Review Board
US: United States
